# Vogt-Koyanagi-Harada disease developed during chemotherapy for Hodgkin lymphoma: a case report

**DOI:** 10.1186/s12886-024-03386-w

**Published:** 2024-03-13

**Authors:** Mutsumi Koyama, Euido Nishijima, Takaya Honda, Chizuru Gonmori-Ohta, Takeaki Sasamoto, Katsuyuki Tanaka, Akira Watanabe, Tadashi Nakano, Masaharu Akiyama

**Affiliations:** 1 Department of Ophthalmology, The Jikei University School of Medicine, 3-25-8, Nishi-shinbashi, Minato-ku, 105-8561 Tokyo, Japan; 2 Department of Pediatrics, The Jikei University School of Medicine, 3-25-8, Nishi-shinbashi, Minato-ku, 105-8461 Tokyo, Japan

**Keywords:** Hodgkin lymphoma, Child, Uveitis, Vogt-Koyanagi-Harada disease

## Abstract

**Background:**

Ocular manifestations are known for non-Hodgkin lymphoma, but are rare for Hodgkin lymphoma. We report a case of Vogt-Koyanagi-Harada (VKH) disease presenting as serous retinal detachment and uveitis in both eyes in a child undergoing chemotherapy for Hodgkin lymphoma.

**Case presentation:**

The patient was a 7-year-old boy with stage IIB Hodgkin lymphoma (nodular lymphocyte predominant type) who was undergoing chemotherapy, including 2 cycles of the OEPA regimen and 1 cycle of the COPDAC regimen. Two days after the end of the COPDAC regimen, the patient complained of headache and of blurred and decreased vision in both eyes. On the basis of optic symptoms, such as uveitis and serous retinal detachment in both eyes, increased cell counts in cerebrospinal fluid, and positivity for human leukocyte antigen (HLA)-DR4 in peripheral blood cells, incomplete VKH disease was diagnosed. Intravenous treatment with high-dose prednisolone (60mg/m^2^/day) for 7 days improved both visual acuity and serous retinal detachment and enabled the remains of the COPDAC chemotherapy cycle to be administered. With prednisolone treatment, visual acuity improved from 20/500 to 20/20 in the right eye and from 20/63 to 20/25 in the left eye. Because multiple vitiligo lesions later appeared in the abdomen, complete VKH disease was finally diagnosed.

**Conclusion:**

The onset of VKH disease occurred during chemotherapy for Hodgkin lymphoma. The patient was HLA-DR4-positive and might have had a predisposition to develop autoimmune diseases, including VKH disease. However, the anticancer drugs administered to this patient have not been reported to cause uveitis. Whether Hodgkin lymphoma triggered the development of VKH remains unclear. Early diagnosis of VKH disease and prompt treatment with high-dose prednisone enabled the patient to maintain good visual function despite chemotherapy for Hodgkin lymphoma.

## Background

Ocular manifestations are known for non-Hodgkin lymphoma, but are rare for Hodgkin lymphoma. An ocular manifestation is uveitis, which is a general term for inflammatory diseases that occur in uveal tissue, such as the iris, ciliary body, and choroid, and in the adjacent retina. Uveitis in persons under 18 years or younger has been reported to account for 2.6–6% of all cases of uveitis in Japan [1, 2]. When classifiable uveitis is divided into 4 etiologies: infectious, noninfectious, paraneoplastic syndrome, and masquerade syndrome [3]. The noninfectious form is the most common, with major causes being sarcoidosis and Vogt-Koyanagi-Harada (VKH) disease in Japan. Reported causes of the infectious form include human herpes virus and cytomegalovirus. Masquerade syndrome is most often caused by non-Hodgkin lymphoma in adult patients. We report a case of VKH disease, presenting as serous retinal detachment and uveitis in both eyes, in a young boy undergoing chemotherapy for Hodgkin lymphoma. We address the pathogenesis and treatment of VKH disease that has developed during chemotherapy for Hodgkin lymphoma.

### Case presentation

A 7-year-old Japanese boy was referred to our hospital because of painless swelling of the bilateral inguinal lymph nodes which had gradually increased for 7 months. His past medical history included atopic dermatitis, and his family history included type 1 diabetes mellitus in his mother. Hematological examination showed the following abnormal values: soluble interleukin-2 receptor, 2,599 U/mL; and immunoglobulin E, 568 IU/mL. A biopsy of a left inguinal lymph node lead to the diagnosis of Hodgkin lymphoma (nodular lymphocyte predominant type). Fluoro-18 fluorodeoxyglucose-positron emission tomography (F-18 FDG PET)/computed tomography (CT) showed a high accumulation (maximum standardized uptake value, SUVmax) at 17.02 in the left common iliac lymph node, left external and internal iliac nodes, and left inguinal lymph node. On the basis of the F-18 FDG PET/CT results and B symptoms, such as weight loss and night sweats, the final staging was determined to be IIB. The patient underwent chemotherapy according to the protocol of the EuroNet-PHL-C1 protocol [4] with 2 cycles of the OEPA regimen (vincristine, etoposide, prednisone, and doxorubicin) and 2 cycles of the COPDAC regimen (cyclophosphamide, vincristine, prednisone, and dacarbazine). Chemotherapy was to be followed by radiotherapy at a dose of 19.8 Gy (11 fractions of 1.8 Gy per day) focused on residual lymph node lesions.

**Fig. 1:**
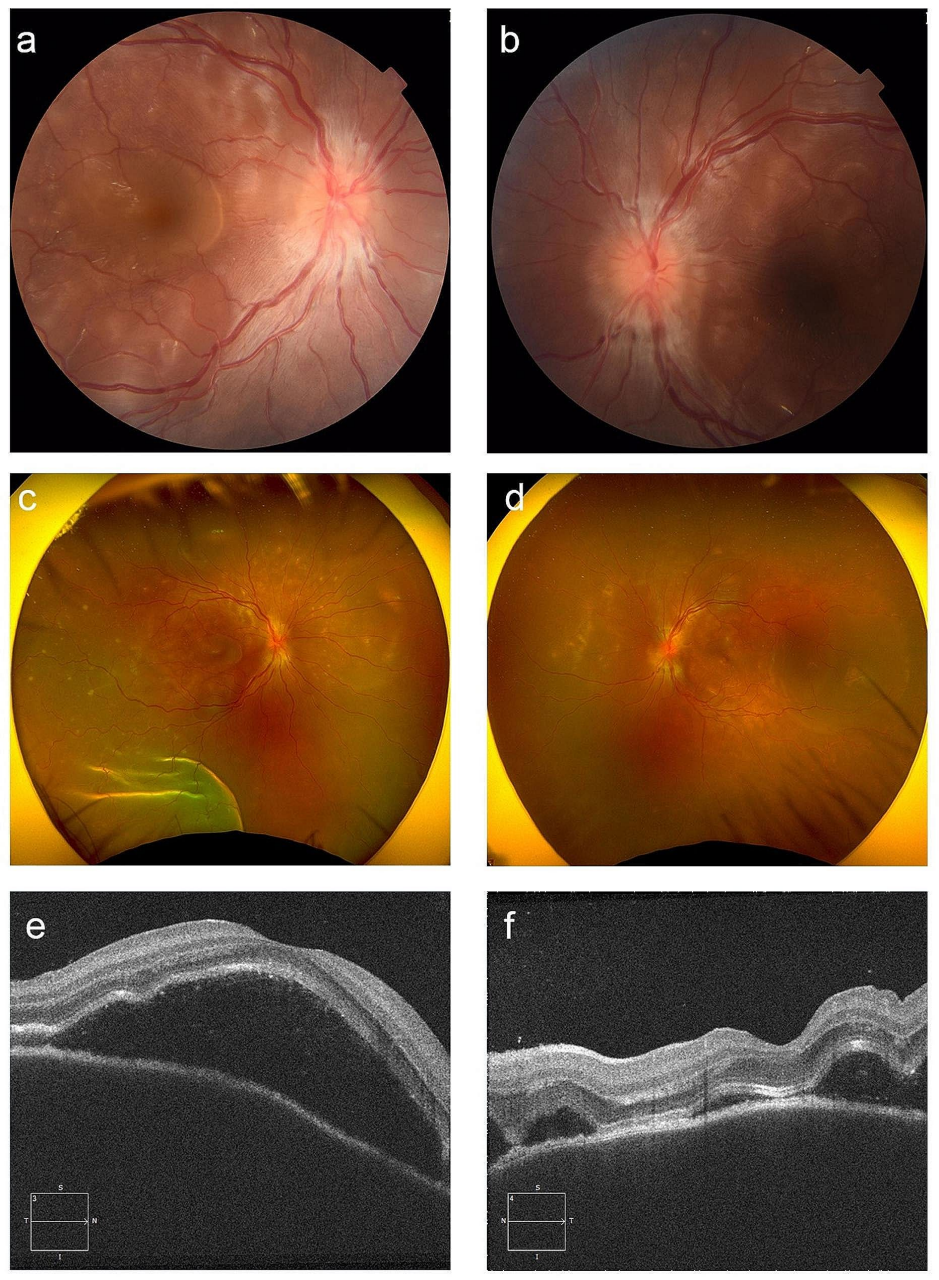
Examination findings at the initial visit. Fundus photograph findings included swelling of the optic nerve papillae in the right eye (**a**) and left eye (**b**) and serous retinal detachment in the right eye (**c**) and left eye (**d**). Optical coherence tomography showed prominent serous retinal detachment in the macular area (**e**, **f**)

**Fig. 2:**
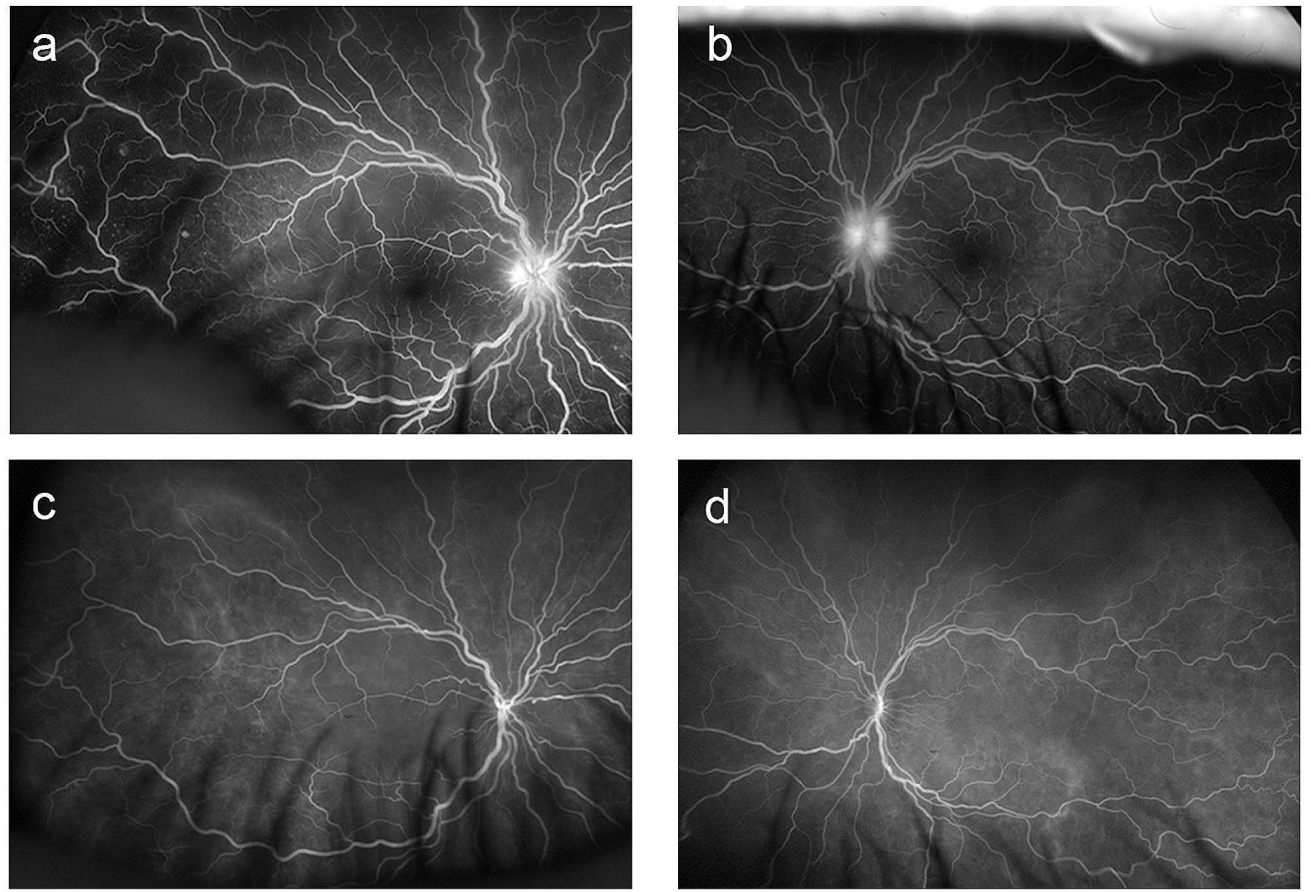
Fundus fluorescence angiography images. The early phase of fluorescein angiography showed fluorescent leakage from the optic nerve papilla and petechial fluorescent leakage in the right eye (**a**) and left eye (**b**); early phase of indocyanine green angiography showed low fluorescent spots in the right eye (**c**) and left eye (**d**)

Two days after the end of the first cycle of the COPDAC regimen, the patient complained of headache and of blurred and decreased vision in both eyes. The first ophthalmologic examination showed a corrected visual acuity of 20/32 in the right eye and 20/40 in the left eye. Intraocular pressure could not be measured in the right eye and was 15 mmHg in the left eye. The anterior chamber depth was normal, with no evidence of inflammatory cells in the anterior chamber. Posterior ocular findings showed swelling of the optic nerve papillae in both eyes (Fig. 1, a, b) and serous retinal detachment. (Fig. 1, c, d). Optical coherence tomography (OCT; Carl Zeiss Meditec AG) showed significant serous retinal detachment in the macular area (Fig. 1, e, f) and significant swelling of the optic nerve papillae in both eyes. Fluorescein angiography of the fundus showed fluorescent leakage from the optic nerve papilla in the early phase and petechial fluorescent leakage in both eyes (Fig. 2, a, b). Indocyanine green angiography showed hypofluorescent dark dots in both eyes in the early phase (Fig. 2, c, d). Ruled out as causes of a possible infectious uveitis via immunological examination were hepatitis B, hepatitis C, syphilis, and human T lymphotropic virus type 1, and ruled out via DNA analysis were herpes simplex virus, varicella-zoster virus, and cytomegalovirus. Cerebrospinal fluid examination revealed an increased cell count. Moreover, human leukocyte antigen (HLA)-DR4 was positive. Because the patient fulfilled items 1 to 4 of the revised diagnostic criteria proposed by the International Workshop on VKH [5], incomplete-type VKH disease was diagnosed.

To treat the VKH disease, the patient received steroid treatment with intravenous prednisolone 60 mg/m^2^/day for 7 days. After improvements in visual acuity and serous retinal detachment were confirmed, 1 cycle of the COPDAC regimen with the dosage increasing from 40 mg/m^2^/day to 60 mg/m^2^/day was started. Prednisolone (60 mg/m^2^/day) was administered for a total of 20 days. Thereafter, the administration of prednisolone was changed from intravenous to oral, after which the dose was gradually tapered. The minimum visual acuity before treatment was 20/400 in the right eye and 20/63 in the left eye. After 22 days of treatment with prednisolone, the visual acuity had improved to 20/20 in the right eye and 20/25 in the left eye, and the intra-anterior chamber inflammation and serous retinal detachment in the macula had disappeared. The steroid treatment was changed to hydrocortisone on day 65 after starting prednisolone. However, anterior chamber inflammation appeared on day 82 of steroid treatment, so the steroid treatment was changed to the dosage increased oral prednisolone again and dexamethasone eye drops were started. Subsequently, the flare-up of inflammation disappeared, and oral prednisolone and dexamethasone eye drops were discontinued.

**Fig. 3:**
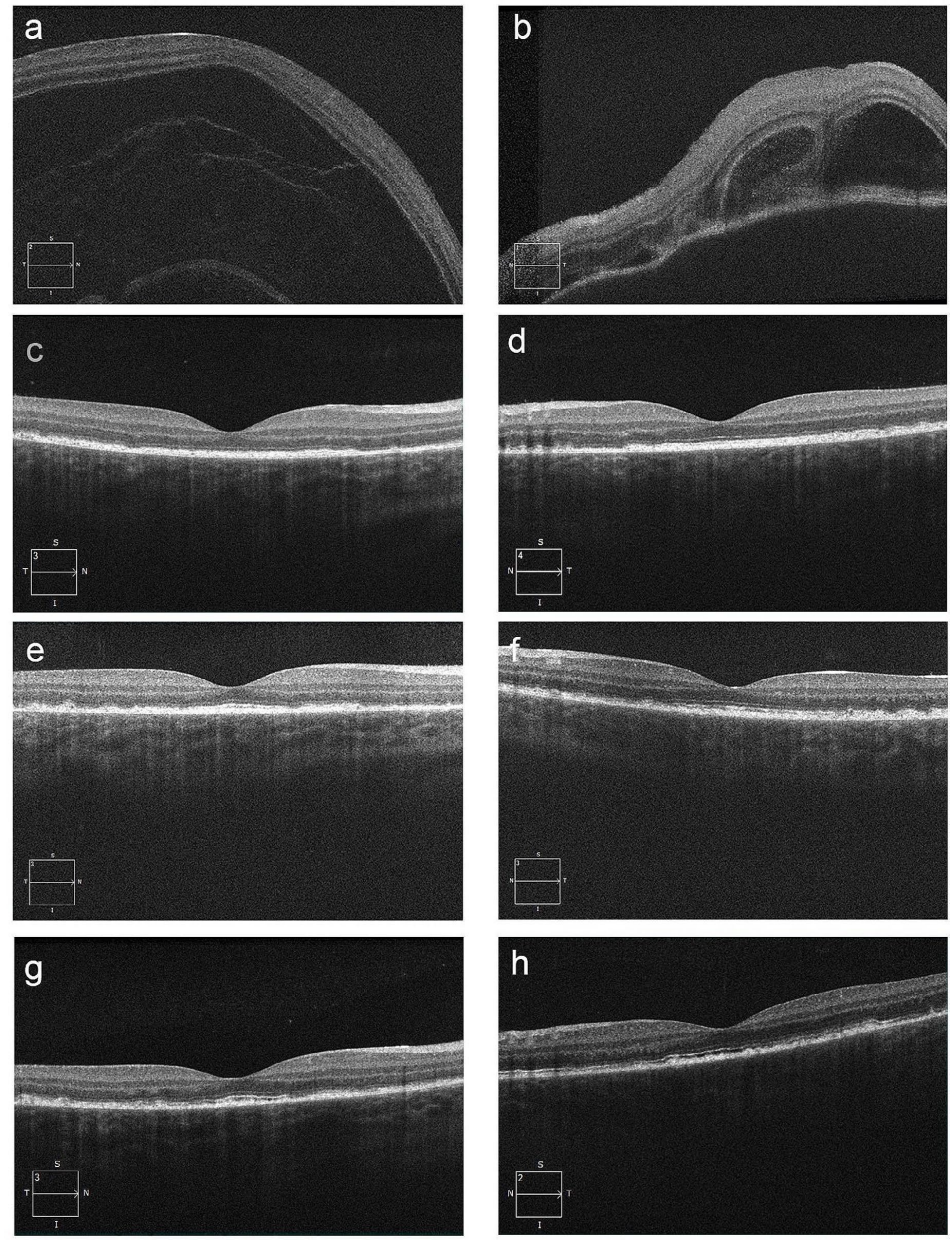
Spectral-domain optical coherence tomography images of the macula. Images of the right eye (**a**: pretreatment, **c**: treatment day 22, **e**: treatment day 43, **g**: treatment day 82) and of the left eye (**b**: pretreatment, **d**: treatment day 22, **f**: treatment day 43, **h**: treatment day 82). Before treatment (**a**, **b**), there was marked serous retinal detachment, which resolved on day 22 (**c**, **d**); on day 43 (**e**, **f**), there were irregular retinal pigment epithelial cells, although visual acuity had improved. On day 82 (**g**, **h**), when the intravitreal inflammation flared up, the irregularity of the retinal pigment epithelial cells worsened, and the irregularity persisted

The findings of OCT changed over the course of treatment (Fig. 3). Serous retinal detachment was marked before treatment (Fig. 3, a, b), but had disappeared after 21 days of steroid treatment (Fig. 3, c, d). On day 43 of steroid treatment, visual acuity had improved, but irregular retinal pigment epithelial cells were observed (Fig. 3, e, f). On day 82, when the intracameral inflammation flared up, the irregularity of retinal pigment epithelial cells worsened and continued (Fig. 3, g, h).

One year after the diagnosis of Hodgkin lymphoma and 9 months after the diagnosis of VKH disease, multiple vitiligo appeared in the patient’s abdomen, and complete VKH disease was finally diagnosed. However, a sunset glow fundus was seen, but Sugiura sign was not. The patient has been doing well and has improved visual function, without evidence of recurrent Hodgkin lymphoma and VKH disease for 10 months after treatment with prednisolone had discontinued.

## Discussion and conclusion

Cases of VKH disease as a complication of Hodgkin lymphoma are rare; few cases have been reported in adults [6, 7]. To our knowledge, the present case is the first to be reported in a child with Hodgkin lymphoma. In the present patient, serous retinal detachment and uveitis in both eyes were observed after completion of 2 cycles of the OEPA regimen and 1 cycle of the COPDAC regimen to treat Hodgkin lymphoma. Complete VKH disease was finally diagnosed according to the diagnostic criteria proposed by the First International Workshop on VKH disease [5].

The believed cause of VKH disease is an autoimmune disease against melanin pigment cells [8]. The disease is caused by an attack on melanin pigment cells, resulting in inflammation of melanin-rich tissues, such as the eyes, ears, meninges, skin, and hair. The immune response to melanin pigment cells is of unknown cause, but is suggested to be related to histocompatibility antigens in leukocytes [8]. Our patient had HLA-DR4, which was a likely predisposition to the development of autoimmune diseases, including VKH disease. One year after Hodgkin lymphoma treatment had been completed, multiple vitiligo appeared on the skin of the abdomen.

Possible causes of the development of VKH disease, other than autoimmune diseases, include paraneoplastic syndrome, masquerade syndrome, infection, and drug-related causes [9]. In the present case, paraneoplastic syndrome could not be ruled out as a possible cause because serum autoantibody testing for retinal antibodies was not performed. Masquerade syndrome is a common term used when the symptoms and findings of the underlying disease are similar to those of other diseases, meaning that the underlying disease is hidden under a mask. The most common causative diseases of uveitis masquerade syndrome are primary intraocular lymphoma [3, 10, 11]. The intraocular lymphomas most often reported are non-Hodgkin lymphomas of histologic type large B-cell lymphoma. The present patient had a Hodgkin lymphoma (nodular lymphocyte predominant type) of the left inguinal region, without findings to suggest bilateral intraocular or central nervous system metastasis. Vitreous opacity and subretinal exudative lesions seen in intraocular lymphomas were not observed. Although vitrectomy and the collection of tumor cells are required to diagnose intraocular lymphoma, we considered the possibility of this disease in our case to be low. Moreover, known causes of VKH disease include such infections as human papilloma virus, bacteria, fungi, cytomegalovirus, and toxoplasma [3], but none of them were detected in the patient. The patient had previously been infected with Epstein-Barr virus, but no evidence of viral reactivation was found during chemotherapy. Furthermore, although drug-related uveitis can be caused by immune checkpoint inhibitors [12], uveitis has not been reported to be caused by any of the antitumor medications administered to patients before VKH disease develops.

During its acute phase, VKH disease in children is generally treated with corticosteroids, but a standard dose has not been established [13]. In the present patient, the treatment of VKH disease overlapped in time with the COPDAC regimen for Hodgkin lymphoma. Because the COPDAC regimen included oral prednisolone at 40 mg/m^2^/day daily for 15 days, the patient was treated with intravenous prednisolone at an increased dose of 60 mg/m^2^/day for 20 days. Thereafter, prednisolone was switched from intravenous to oral administration, and then the dose was gradually decreased. However, intraocular inflammation had been worse when the prednisolone dose had been reduced to avoid adverse effects, such as suppression of adrenal function and growth retardation, suggesting that the rate of prednisolone should be noted.

The onset of VKH disease occurred during chemotherapy for Hodgkin lymphoma. The patient was HLA-DR4-positive and might have had a predisposition to develop autoimmune diseases, including VKH disease. However, anticancer drugs administered to this patient have not been reported to cause uveitis. Whether Hodgkin lymphoma triggered the development of VKH remains unclear. Early diagnosis of VKH disease and prompt treatment with high-dose prednisone for an extended period enabled the patient’s visual function to be well maintained. The establishment of dosage and duration of corticosteroids administration for treating VKH disease in patients with underlying diseases is challenging, and additional cases must be reported.

## Data Availability

Data sharing is not applicable to this article as no datasets were generated or analyzed during the current study.

## Abbreviations

CT: Computed tomography
F-18 FDG PET: Fluoro-18 fluorodeoxyglucose-positron emission tomography
HLA: Human leukocyte antigen
VKH: Vogt-Koyanagi-Harada
